# Morphological and molecular data reveal a new genus of the tribe Issini from Southern China (Hemiptera, Fulgoromorpha, Issidae)

**DOI:** 10.3897/zookeys.766.24299

**Published:** 2018-06-13

**Authors:** Menglin Wang, Aimin Shi, Thierry Bourgoin

**Affiliations:** 1 Key Laboratory of Southwest China Wildlife Resources Conservation of the Ministry of Education, China West Normal University, Nanchong,; 2 Sichuan Province, 637009, China; 3 Institut de Systématique, Évolution, Biodiversité, ISYEB-UMR 7205 MNHN-CNRS-Sorbonne Université-EPHE, Muséum national d’Histoire naturelle, CP 50, 57 rue Cuvier, F-75005 Paris, France

**Keywords:** China, new species, *Sinonissus*, taxonomy

## Abstract

A new genus *Sinonissus* **gen. n.** of the tribe Issini (Issidae, Issinae) with a new species *Sinonissus brunetus* **sp. n.** from Chongqing municipality and Sichuan Province, China are described. Barcode of the species is provided. A molecular analysis combined with morphological characters confirms its placement into the Issini. Distribution of this new genus in the Oriental realm is briefly discussed in regard of other Issinae taxa in China.

## Introduction

In the family Issidae Spinola, 1839 (Hemiptera: Fulgoromorpha), Issini Spinola, 1839 (sec. Wang et al. 2016) is a small planthopper tribe, consisting of only two extant genera: *Issus* Fabricius, 1803 and *Latissus* Dlabola, 1974 with 39 species worldwide (Bourgoin 2018). A third monospecific fossil genus from Middle Eocene in Germany, *Issites glaber* Haupt, 1956 was recently reviewed and added to the tribe (Gnezdilov and Bourgoin 2016).

The lineage was firstly separated as a subtribe Issini Spinola, 1839 by Gnezdilov (2002: 609), later abandoned (Gnezdilov 2016a: 343), but confirmed valid by different molecular analyses (Gnezdilov et al. 2015, Wang et al. 2016) and treated as a separate tribe of Issinae by Wang et al. (2016). In this last treatment, Issini is typically distributed in the Wallace Palaearctic (western area particularly) and Oriental regions, and more precisely into Holt’s (2013) Oriental, Sino-Japanese, Palaearctic, and Saharo-Arabian zoogeographic realms (Gnezdilov et al. 2015, Bourgoin 2018). *Issus* and *Latissus* genera are natively distributed in the Mediterranean area of the Western Palaearctic region (Gnezdilov et al. 2014). They form the “*Issus* group” which is thought to have diverged early from the other Western Palaearctic taxa and was one of the first groups to colonise the proto-Mediterranean communities of the ancient Mediterranean in the Eocene (Gnezdilov 2016a, 2016b).

Issini are characterised by the presence of paired digitate processes on the inner side of the dorsolateral lobes of the periandrium (Gnezdilov 2016a), and not exclusive to the lineage, the veins run in parallel, and according to the schema “R2, M2, CuA2” until the tip of the tegmina (Gnezdilov 2003).

A new genus *Sinonissus* gen. n. is described from southwest China in the Oriental realm, represented by the new species *Sinonissus brunetus* sp. n. from Chongqing and Sichuan, for which both morphological data and molecular phylogeny place the taxon into the Issini sec. Wang et al. (2016).

## Materials and methods

Type specimens are deposited in College of Life Science, China West Normal University, Nanchong, Sichuan Province, China. The abdomen of specimen was separated from the body, and then boiled in a 10% NaOH solution for 5 minutes until muscles were completely dissolved leaving tegumentary structures. After rinsing in distilled water for several times, the abdomen was subsequently transferred to glycerine for final dissection and observation. Terminalia were conserved under the specimen in genital vials. Photographs for external morphology and terminalia characters were taken using Leica DFC495 camera attached to Leica M205C stereomicroscope and further refined with LAS V3.8 and Helicon Focus v3.10 software. Morphological interpretations and subsequent terminologies for male genitalia follow Bourgoin (1987), for female genitalia Bourgoin (1993) and for wing venation Bourgoin et al. (2015).

The total genomic DNA was extracted from leg of holotype specimen (♂) using the TransGen EasyPure Genomic DNA Kit. COI gene was amplified using the same primers and amplification procedure as Wang et al. (2016). The DNA sequencing was conducted at Sangon Company (Shanghai, China). Software Seqman from package DNAstar v5.01 (www.dnastar.com) was used for checking sequence chromatograms and assembling contigs. Mega v7.0 (Kumar et al. 2016) was used for performing alignments. IQtree v1.4.1 (Nguyen et al. 2015) was used for maximum likelihood phylogenetic analysis using 10000 ultrafast bootstrap (Minh et al. 2013) with substitution model automatic selected. Figtree v1.1.2 (Rambaut 2016) was used to visual the tree. The COI sequence of *Sinonissus brunetus* sp. n. was registered in GenBank with accession number MG921598, the other COI sequences used in this study were obtained from Wang et al. (2016).

## Taxonomy

### Issidae Spinola, 1839

#### Issinae Spinola, 1839

##### Issini Spinola, 1839

###### Sinonissus gen. n.

Taxon classificationAnimaliaHemipteraIssidae

http://zoobank.org/144A599D-DD5E-403A-AE46-95F43B9CAAC5

####### Type species.

*Sinonissus brunetus* sp. n., here designated.

####### Diagnosis.

This genus is similar to the genus *Latissus* Dlabola, 1974 (Gnezdilov et al. 2011, fig. 4; Gnezdilov et al. 2014, figs 13d–f) in general appearance, but differs by: 1) vertex without median carina but with carina in *Latissus*; 2) tubercles on frons very tiny and obscure but large and elevated in *Latissus*; 3) Pcu and A_1_ fused at basal half of clavus but fused at apical 1/3 in *Latissus*.

####### Description.

**Figures 1–3.**
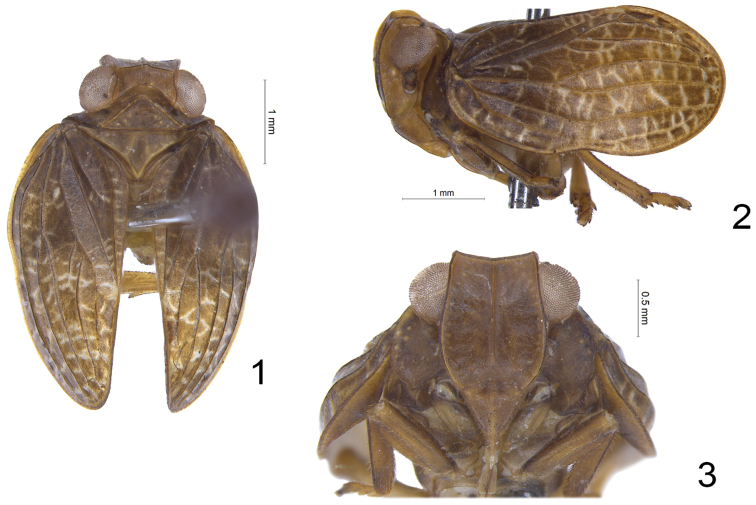
*Sinonissus brunetus* sp. n., holotype. **1** Adult, dorsal view **2** Adult, lateral view **3** Adult, ventral view.

Head with compound eyes a little wider than pronotum, but nearly the same width as mesonotum (Fig. 1). Vertex rectangular, obviously broader than long, anterior margin elevated, slightly convex or nearly straight, lateral margins elevated, apical half nearly parallel and basal half broaden outward (Fig. 1) or parallel all the time, posterior margin anteriorly widely concave at middle, median carina absent on disc (Fig. 1). Frons obviously longer than wide, slightly broaden below level of compound eyes (Figs 3, 16); apical margin slightly concave almost straight, apical and lateral margins carinate and elevated, median carina elevated from apex extending to near base, but not reaching frontoclypeal sulcus (Figs 3, 16); frons with lateral area distributed with some faint tiny tubercles (Fig. 3). Frontoclypeal suture strongly convex (Fig. 3). Clypeus with median carina (Fig. 3). Rostrum slightly exceeding mesocoxae, apical segment shorter than subapical one. Gena in lateral view slightly protrude below frontoclypeal suture (Fig. 2). Antenna with scape short and cylindrical, pedicel rounded. Pronotum triangular, margins elevated, with several indistinct tubercles on disc, median carina absent (Fig. 1). Mesonotum with two carinae on the disc (Fig. 1). Forewings ovate, longitudinal veins obvious and elevated, costal margin and posterior margin subparallel (Figs 2, 15), with wide ‘hypocostal plate’ (Gnezdilov 2003) (Fig. 3), short common stem ScP+R separating in unforked ScP+RA and RP and reaching the outer margin of forewing; MP forking only once near the basal 1/3 into unforked MP_1+2_ and MP_3+4_; CuA forking into CuA_1_ and CuA_2_ near middle (Figs 2, 15). Clavus closed, Pcu and A_1_ fused at basal half of clavus (Figs 1, 15). Hindwing very rudimentary, almost absent. Metatibia with two lateral spines on apical half and approximately eight apically.

**Figures 4–7.**
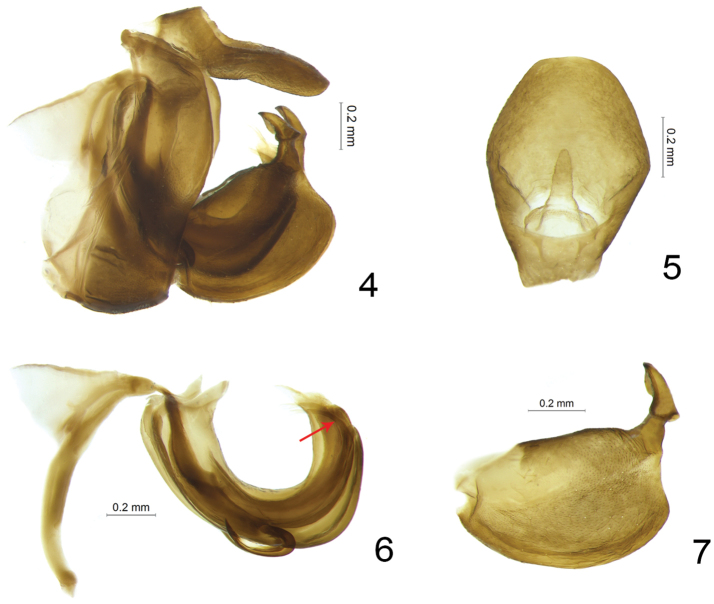
*Sinonissus brunetus* sp. n., holotype. **4** Male terminalia, lateral view **5** Male anal tube, dorsal view **6** Phallic complex, lateral view **7** Gonostylus, lateral view. The red arrow indicates the paired digitate processes on the dorsolateral lobe of periandrium.

**Male terminalia.** Gonostyli subrectangular in profile, caudo-ventral angle rounded, dorsal margin without process (Figs 4, 7); capitulum broad, with auricular process (Fig. 7). Pygofer rectangular in lateral view, apparently longer than wide (Fig. 4). Periandrium symmetrical, tubular, apical part divided into dorsolateral lobe and ventral lobe (Figs 6, 17). Aedeagus with the pair of aedeagal processes emerging at 3/5 of periandrium length, hook-like, short (Figs 6, 17).

**Figures 8–14.**
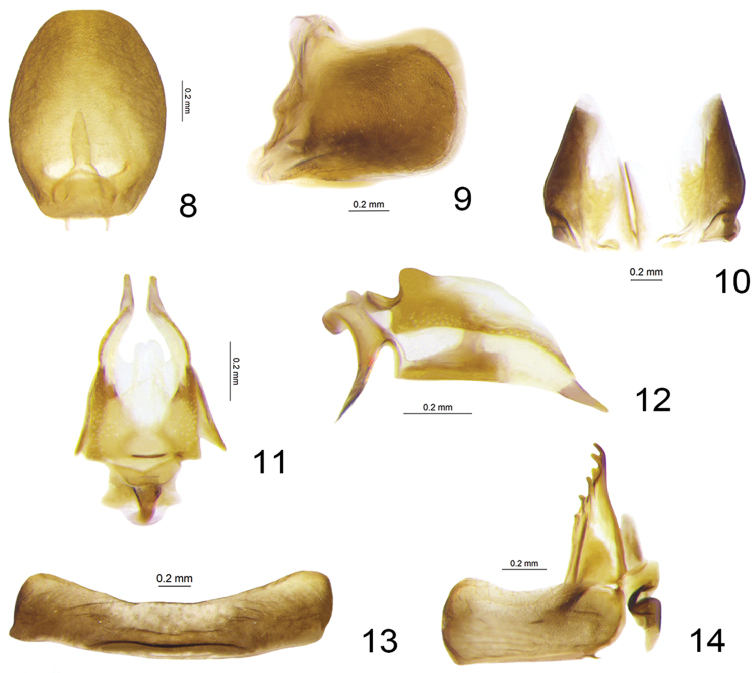
*Sinonissus brunetus* sp. n., paratype. **8** Female anal tube, dorsal view **9** Gonoplacs, lateral view **10** Gonoplacs, dorsal view **11** Gonapophysis IX and gonaspiculum bridge, dorsal view **12** Gonapophysis IX and gonaspiculum bridge, lateral view **13** Sternite VII **14** Gonocoxa VIII and gonapophysis VIII, lateral view.

**Female terminalia.** Anal tube relatively short, in dorsal view a little longer than wide (Fig. 8). Two or three teeth at apex and three keeled teeth on outer lateral margin of anterior connective lamina of gonapophysis VIII (Fig. 14). Gonocoxa VIII quadrangular, connected to gonapophysis VIII with rectangular angle (Fig. 14). Gonapophysis IX in lateral view boat-shaped (Fig. 12). Gonoplacs rectangular in lateral view (Fig. 9), fused at middle near base, widest at basal 1/3 (Fig. 10). Hind margin of sternite VII concave medially (Fig. 13).

**Figures 15–18.**
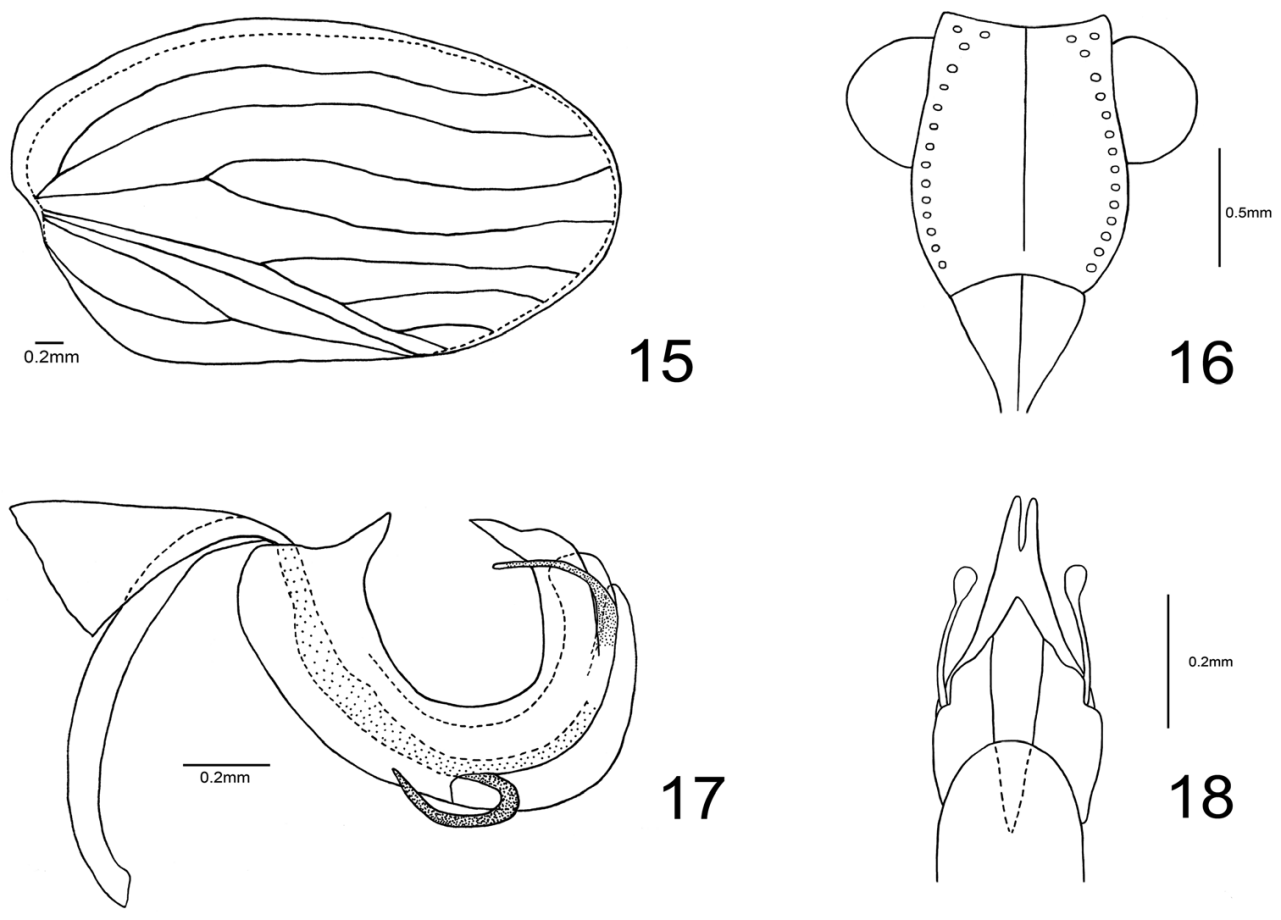
*Sinonissus brunetus* sp. n. **15** Forewing **16** Frons **17** Phallic complex, lateral view **18** Apex of phallic complex, ventral view.

####### Distribution.

China (Chongqing, Sichuan).

####### Etymology.

This name is derived from the Latin prefix word “*sino*” freely associated with the generic name “*Issus*”, referring to the special distribution of this genus representing the rarity of Issini in China. The gender is masculine.

####### Remarks.

**Figure 19.**
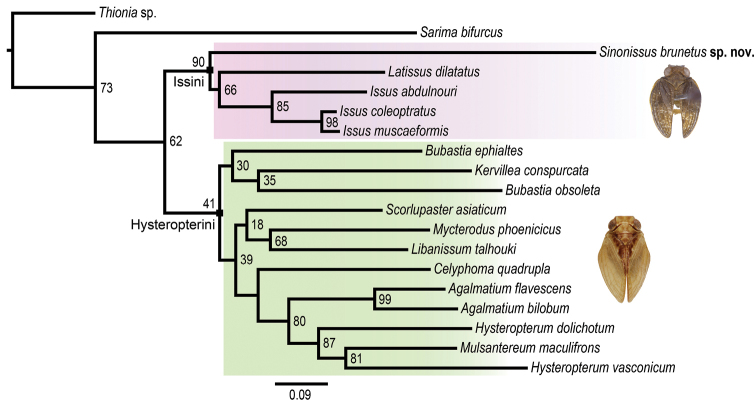
Maximum likelihood tree of Issinae based on COI sequence with *Thionia* sp. (Issidae Thioniinae) and *Sarima bifurcus* (Issidae, Hemisphaeriinae) as outgroup to test to position of *Sinonissus* gen. n. in the classification and phylogeny of Issidae. Node values denote ultrafast bootstrap support.

The new genus differs from *Issus* by the presence of a wide hypocostal plate, also present in *Latissus*, and from both genera by its rudimentary hindwings. It shows that this last character is not characteristic of the tribe, for which the diagnosis should be modified accordingly. Additionally, the Issini forking schema ‘R2, M2, CuA2’ indicating the number of main vein terminals in the tegmen appears to be modified in *Sinonissus* with two to four terminals in CuA: accordingly a ‘R2, M2, CuA(2–4)’ schema should be retained for Issini sec. Wang et al. (2016)’s diagnosis. In reverse, *Sinonissus* shares particularly with them the presence of paired digitate processes on the dorsolateral lobes of periandrium, two lateral and 5–9 apical metatibial spines. Molecular phylogeny analysis confirms also the placement of the taxon as sister to the two others, according to the schema (*Sinonissus* + (*Latissus* + *Issus*)) with node value of 90 (Fig. 19).

###### Sinonissus brunetus sp. n.

Taxon classificationAnimaliaHemipteraIssidae

http://zoobank.org/710BAE8A-F0F7-4DDA-BA4F-30FBDAF30A42

Figs 1–3 , 4–7 , 8–14 , 15–18

####### Type materials.

Holotype: ♂, China: Chongqing municipality, Jinyunshan, 6 vii 2017, coll. Menglin Wang. Paratypes: 1♂, Chongqing municipality, Jinyunshan, 5 vii 2011, coll. Ting Xu; 1♀, Sichuan Province, Emeishan, 5 vii 2010, coll. Meiyi Xia; 1♀, Sichuan Province, Emeishan, 5 vii 2010, coll. Yuling Zhang.

**Diagnosis.** This new species looks similar to *Latissus dilatatus* (Fourcroy, 1785), but differs by: frons much longer, 1.2 times longer in midline than broad at widest part (only 0.9 times in *L. dilatatus*); anal tube of male 1.4 times longer in midline than widest part (2.2 times in *L. dilatatus*); male genitalia less robust, the digitate processes near apex of periandrium slender and curved (broad and straight in *L. dilatatus*).

####### Description.

Length: male (including forewings) (N = 2): 4.2–4.3 mm; female (including forewings) (N = 2): 6.2–6.3 mm.

**Coloration.** Vertex brown, margins carinated and dark brown (Fig. 1). Compound eyes dark grey, supported by tawny callus (Fig. 1). Frons brown, apical and lateral margins carinated and dark brown, brown median carina extending from apex near to base, but not reaching the frontoclypeal sulcus (Figs 3, 16); lateral area of frons with some tawny inconspicuous tubercles on each side near the lateral margins (Figs 3, 16). Postclypeus brown, rostrum light brown (Fig. 3). Gena brown (Fig. 2). Antennae dark brown (Fig. 3). Pronotum brown, margins carinated and dark brown, lateral area with three unconspicuous light yellow tubercles on each side (Fig. 1). Mesonotum brown, lateral carinae dark yellow (Fig. 1). Forewings brown, longitudinal veins dark brown and transverse veins grey (Figs 1, 2). Legs brown (Figs 2, 3).

**Head and thorax.** Vertex 3.1 times wider at base than long in midline, lateral margins parallel in apical 1/2 and expanded outward at basal 1/2 (Fig. 1) or parallel all the time. Frons 1.2 times longer in midline than broad at widest part, 1.3 times broader at widest part than apical margin (Fig. 3). Pronotum 2.3 times wider at base than long in midline, anterior margin angularly convex, lateral margins straight (Fig. 1). Mesonotum with anterior margin 2.4 times wider than long in midline (Fig. 1). Forewings 1.9 times wider at longest part than widest part. Metatibiotarsal formula: 2–(7–8)/(6–8)/2.

**Male terminalia.** Anal tube in dorsal view ovate, widest at apical 1/3; 1.4 times longer in midline than widest part, apical part rounded; epiproct long, around 1/3 length of anal tube, anal opening located at basal 1/3 (Fig. 5). Gonostylus subrectangular in lateral view, dorsal margin straight and sloping up posterior, posterior margin nearly straight, caudo-ventral angle rounded, and ventral margin rounded (Figs 4, 7). Capitulum of gonostylus relatively short and broad with an auriform process in the apical 2/3 (Figs 4, 7). Pygofer in lateral view much longer than broad, dorsal margin inclined downward, anterior and posterior margins sinuate (Fig. 4). Periandrium with dorsolateral lobe relatively triangular, weakly sclerotised, ventral lobe rounded apically in lateral view (Figs 6, 17); dorsolateral lobe longer than ventral lobe; pair of slender slightly sclerotised digitate processes originated from the dorsolateral lobe near the apex, curved upward and directed cephalad (Figs 6, 17). In ventral view apical part of dorsolateral lobe sharp, apical margin of ventral lobe rounded (Fig. 18). Paired aedeagal processes hooks-like, curved upward, originated from the basal 3/5 of phallic complex extending to the basal 2/5, tip of processes pointed and directed to dorso-anterior part (Figs 6, 17).

**Female terminalia.** Anal tube in dorsal view ovate, widest at middle, 1.2 times longer in midline than widest part, apical margin and lateral margins rounded; epiproct long, approximately 1/3 length of anal tube, anal opening situated at basal 1/4 (Fig. 8). Anterior connective lamina of gonapophysis VIII with two or three teeth at apex and three keeled teeth on the outer lateral margin, inner lateral margin without teeth (Fig. 14). Endogonocoxal process developed, slightly sclerotised in basal half and membranous in distal one (Fig. 14), apex of endogonocoxal process with two-digitate processes. Posterior connective lamina of gonapophysis IX in lateral view long and narrow, boat-shaped, tip pointed, dorsal margin roundly convex at base (Fig. 12); in dorsal view basal half broader than apical half, the apical half narrower to apex in outer lateral margins, bifurcate at apical 1/3 in inner part, basal half with outer margins nearly parallel, lateral area sclerotised (Fig. 11). Gonospiculum bridge small and short, in lateral view rectangular with needle-like ventrally (Fig. 12). Gonoplacs fused near base, outer lateral margins roundly convex (Fig. 10), in lateral view rectangular (Fig. 9).

####### Etymology.

The Latin name *brunetus*, referring to the dark brown colour of the general appearance of this species.

####### Distribution.

China (Chongqing, Sichuan).

####### Remarks.

The COI nucleotide composition of this species is A: T: G: C = 32.7: 32.7: 14.5: 20.0. It differs by 124 and 126 nucleotidic bases with *Issus coleoptratus* (Fabricius, 1781) (GenBank accession number: KX702932) and *Latissus dilatatus* (Fourcroy, 1785) (GenBank accession number: KX702947) respectively, along the complete length of 681 bp.

## Discussion

The sub-family Issinae currently includes two tribes: Issini and Hysteropterini . They are characteristically distributed in Wallace’s Palaearctic region (Bourgoin 2018), with exceptional distributions from Palaearctic Africa (= Saharo-Arabian Holt’s 2013 realm) to Afrotropical or Oriental regions (= Sino-Japanese and Oriental Holt’s 2013 realm). However, most true Issinae’s non-Palaearctic occurrences need confirmation as these records are based on old observations, probably misidentified, or still not formally correctly assigned to the correct tribe (e.g., *Eupilis* Walker, 1857 in the Hysteropterini was shown to be close to *Tempsa* in the Sarimini by Gnezdilov 2016c).

In China, Issinae are rare but Hysteropterini were already reported from Xinjiang, Ningxia, Qinghai, Inner Mongolia and Gansu: *Celyphoma* Emeljanov, 1971 by Meng and Wang (2012), Chen et al. (2014) and Sichuan: *Hysteropterum boreale* = *Potaninum boreale* (Melichar, 1902) by Gnezdilov (2017). Issini have also been reported from Hong Kong (*Issus quadriguttatus* Walker, 1851 = *Issus coleoptratus* (Fabricius, 1781) by Gnezdilov et al. (2004). *Sinonissus* is therefore the second genus of the Issini to be reported from China. It appears to be a new genus of this rare group of taxa which has crossed Palaearctic and Sino-Japanese realm frontiers to evolve into the Oriental realm in the southwest areas of Chongqing and Sichuan in China.

Gnezdilov (2016a: 333 and fig. 43) supposed that the Issini lineage (sec. Gnezdilov, 2002 = Issini sec. Wang et al. 2016) had diverged early in the tree of the Western Palaearctic taxa as sister taxa to all other western Palaearctic genera, being one of the first groups to colonise the proto-Mediterranean communities of the ancient Mediterranean in the Eocene (Gnezdilov 2016b). The discovery of *Sinonissus* in the Issini lineage shows that radiation of Issinae in the Palaearctic is probably more complex than expected. Most Issinae genera still need to be molecularly tested to enable their possible placement in the phylogeny and is probably that other genera should join this tribe Issini to fill gaps in this paradoxical geographical distribution.

## Supplementary Material

XML Treatment for Sinonissus

XML Treatment for Sinonissus brunetus
